# GSDME Increases Chemotherapeutic Drug Sensitivity by Inducing Pyroptosis in Retinoblastoma Cells

**DOI:** 10.1155/2022/2371807

**Published:** 2022-03-29

**Authors:** Fang Li, Qinyun Xia, Lian Ren, Yuhong Nie, He Ren, Xiaoyu Guo, Jinqiang Yu, Yiqiao Xing, Zhen Chen

**Affiliations:** ^1^Eye Center, Renmin Hospital of Wuhan University, Wuhan, Hubei, China; ^2^Department of Ophthalmology, Renmin Hospital, Hubei University of Medicine, Shiyan, Hubei, China; ^3^Zhongnan Hospital of Wuhan University, Institute of Hepatobiliary Diseases of Wuhan University, Transplant Center of Wuhan University, Hubei Key Laboratory of Medical Technology on Transplantation, Wuhan, Hubei, China

## Abstract

Chemotherapy is an important part of retinoblastoma (RB) treatment. However, the development of drug resistance increases the likelihood of treatment failure. Therefore, increasing the sensitivity of chemotherapeutic drugs is very important. Recent research has explored the relationship between the expression level of gasdermin E (GSDME) and drug sensitivity in RB. Our study found that GSDME expression was significantly reduced in human RB tissues and cell lines. Downregulation of GSDME expression reduced the sensitivity of cells to chemotherapeutic drugs. Decitabine treatment and transfection with GSDME-overexpressing lentivirus (LV-GSDME) upregulated GSDME expression in Y79 and WERI-RB-1 cell lines. The half maximal inhibitory concentrations (IC_50_) for carboplatin-induced cell death were significantly reduced. Low-dose carboplatin could achieve the IC_50_, and no significant difference was found in the production of prodeath-activating proteins, but the mode of cell death changed from apoptosis to pyroptosis. Increased GSDME expression can reduce the required dose of chemotherapeutic drugs. After inhibition of caspase-3 activation, the IC_50_ of carboplatin-induced cell death was significantly increased in cells with high GSDME expression, and the method of cell death switched from pyroptosis to apoptosis, which increased the concentration of chemotherapeutic drugs. Furthermore, the sensitivity of cells to carboplatin was reduced. The *in vivo* xenograft tumor model further confirmed that GSDME upregulation could promote carboplatin-induced tumor cell death. Therefore, the sensitivity of cells to chemotherapeutic drugs can be predicted by detecting the GSDME expression level, and we used pyroptosis induction as a new method for promoting tumor death.

## 1. Introduction

Retinoblastoma (RB) is the most common primary intraocular malignant tumor in children, with a global incidence of 1 : 15,000-1 : 20,000 of the birth rate [1]. Without prompt treatment, RB will lead to death due to metastasis. Therefore, the main goal of treatment is to maintain life, and the secondary goals are to preserve the eyeball, maintain vision, and improve the quality of life [2]. Enucleation is the most important treatment at present, but with the introduction of systemic chemotherapy and local therapy, the use of radiotherapy has been gradually eliminated, and new routes of targeted drug delivery (intra-arterial, intravitreal, and intracameral injection) have been adopted. The advent of *in situ* chemotherapy has markedly improved the rate of eye preservation and reduced the use of systemic chemotherapy [2, 3]. As a result of drug treatments for RB, preserving the eyeballs and vision of an increasing number of patients has become possible.

Advances in chemotherapeutic drugs and chemotherapy methods have significantly reduced the enucleation rate and mortality of RB patients. However, a new problem has emerged: tumors are prone to develop resistance to chemotherapeutic drugs, resulting in treatment failure. Therefore, the discovery of new targets and new mechanisms of chemotherapy for RB is critical. In terms of drug treatments for RB, carboplatin, a common chemotherapeutic drug, mainly functions to cause DNA damage. After entering the cell, carboplatin binds to the N7 reaction center on the purine residue to form crosslinks, thereby damaging the DNA in the cell and further inducing cell apoptosis [4]. However, in tumor cells, due to the mutation of DNA repair enzymes, the crosslinks formed after DNA damage caused by carboplatin are removed and repaired, and apoptosis cannot be induced, which causes tumor cells to develop resistance to chemotherapeutic drugs [5, 6]. Changes in the enzyme system involved in DNA repair and the activity of DNA topoisomerase II have been observed in drug-resistant melanoma cells [7–9]. Therefore, for drug-resistant tumors that cannot induce apoptosis, the discovery of a new death mode that replaces apoptosis has become a new therapeutic approach.

Apoptosis is the most common form of programmed cell death caused by chemotherapeutic drugs. However, there are several forms of cell death in addition to apoptosis, including necrosis, autophagy, and pyroptosis [10]. Apoptosis is spontaneous death under the control of genes. The cell membrane remains intact, and inflammation generally does not occur [11]. Pyroptosis was first discovered in the early 1990s. At that time, some laboratories found that infection with *Shigella flexneri* or *Salmonella enterica* triggered rapid cytotoxic death in mouse macrophages [12, 13]. Moreover, poly (ADP-ribose) polymerase (PARP) activation or DNA fragments are not necessary elements for the rapid lysis characteristic of *Salmonella*-infected macrophages [14]. This new type of programmed inflammatory death is similar to apoptosis. The pyroptotic cells undergo nuclear condensation and chromatin DNA fragmentation, and terminal deoxynucleotidyl transferase dUTP nick-end labelling (TUNEL) staining is positive. Pyroptosis is also similar to necrosis. During the process of cell death, the formation of pores disrupts the balance of the ion gradient on both sides of the cell membrane, leading to the influx of extracellular fluid, cell swelling, cell membrane rupture, the release of proinflammatory mediators, and the induction of the inflammatory response; therefore, pyroptosis is also called inflammatory necrosis [15–17].

Pyroptosis is mediated by the gasdermin (GSDM) family of GSDMA, GSDMB, GSDMC, DFNA5/GSDME, and DFNB59 [18]. GSDME is a member of the GSDM family and can be cleaved by activated caspase-3 to form an active N-terminal domain (GSDME-N), which is inserted into the cell membrane to form pores, causing cell swelling and lysis, the release of inflammatory substances, and pyroptosis [19, 20]. Moreover, GSDME-N can be inserted into the mitochondrial membrane to increase the release of cytochrome C, further expand the activation of caspase-3/7, and increase cell death [21]. Evidence suggests that GSDME is a tumor suppressor with reduced expression in many tumors, mainly due to epigenetic silencing caused by methylation of the promoter region [22, 23]. The use of low-dose decitabine, a methyltransferase inhibitor, can increase GSDME expression and inhibit tumor growth [24]. GSDME can also inhibit the proliferation of gastric cancer, melanoma, and colorectal cancer cells and the invasion of breast cancer [10, 21, 25]. Due to the inhibitory effect of GSDME on tumors, its mechanism of action has been increasingly studied in depth. First, high GSDME expression can convert cells from apoptosis to pyroptosis and can expand the activation of caspase-3/7, further enhancing cell death [21]. Then, GSDME can inhibit tumor growth by activating antitumor immunity [26]. An increasing number of studies have focused on treating tumors with pyroptosis as another means of inducing cell death [5, 27]. Based on the principle of GSDME-induced pyroptosis, we can convert cell death that is resistant to apoptosis into pyroptosis, thereby promoting cell death. In the present work, we answer the following questions: does GSDME expression differ in RB? Can we determine the relationship between tumor drug resistance and GSDME based on the difference in GSDME expression? Can we increase GSDME expression to induce pyroptosis to increase drug sensitivity? Starting with this theory, we further studied GSDME in RB to develop a new treatment for RB patients with drug resistance.

## 2. Material and Methods

### 2.1. Tumor Specimens and Immunohistochemistry

The samples of human RB were obtained from Renmin Hospital of Wuhan University. This study was approved by the Ethics Review Committee of Renmin Hospital of Wuhan University, and informed consent was obtained from all patients. Forty-one RB samples and peritumoral normal retinal tissue were collected from 41 patients who underwent enucleation without chemotherapy and radiotherapy at Renmin Hospital of Wuhan University for immunohistochemistry. The samples were fixed with 10% formalin and embedded in paraffin. The samples were sliced into sections. The sections were deparaffinized in xylene and rehydrated in a descending alcohol series. Antigen retrieval was performed by heating in a pressure cooker in 10 mmol/l citrate buffer. Endogenous peroxidase activity was blocked by incubation in 0.3% H_2_O_2_, followed by incubation with 5% serum to reduce nonspecific binding. The sections were incubated with a primary antibody against GSDME (#13075, Proteintech, China) at 4°C overnight. After washing in phosphate-buffered saline (PBS), the slides were incubated with horseradish peroxidase-conjugated secondary antibody, developed using 3,3-diaminobenzidine (DAB) chromogen solution, and counterstained with Mayer's haematoxylin. A percentage of GSDME was scored on a scale of 0 (no), 1 (1-25%), 2 (26-50%), 3 (51-75%), and 4 (76-100%), and the intensity of GSDME staining was scored on a scale of 0 (no), 1 (weak), 2 (medium), and 3 (strong). The final score was defined as the sum of these parameters.

### 2.2. Cell Culture and Reagents

The human RB cell lines Y79 and WERI-RB-1 (Procell Life Science & Technology Co., Ltd., China) were cultured in the Roswell Park Memorial Institute 1640 (RPMI 1640 (HyClone, USA)), and the normal retinal pigment epithelial cell line ARPE-19 (Procell Life Science & Technology Co., Ltd., China) was cultured in Dulbecco's modified eagle medium (DMEM) (HyClone, USA) and high-glucose medium at 37°C with 5% CO_2_. All media were supplemented with 10% foetal bovine serum (FBS) (Gibco, USA) and 1% penicillin-streptomycin (Bioswamp, China).

### 2.3. Cell Proliferation Assay

Approximately 1000 Y79, 1000 ARPE-19, and 3000 WERI-RB-1 cells were inoculated into 96-well plates and cultured for 24 h. Carboplatin (MedChemExpress, USA) was completely dissolved in 0.9% saline and added to cells at different concentrations, and the difference in volume was balanced with 0.9% saline at the time of addition. Cell viability was detected by Cell Counting Kit-8 (CCK8 (Vazyme, China)) at 24 h, 48 h, and 72 h. Forty-eight hours was found to be the best time for carboplatin to inhibit cell growth, 24 h was too short to achieve the half maximal inhibitory concentrations (IC_50_), and 72 h was too long. The consumption of nutrients in the medium and accumulation of metabolic waste caused extensive cell death in the normal group, leading to errors in the results. Therefore, all subsequent tests were performed after 48 h of carboplatin intervention, except for flow cytometry at 24 and 48 h of carboplatin intervention.

### 2.4. RNA Isolation and Real-Time PCR Analyses

The cells were cleaned with PBS twice, and RNA was extracted using TRIzol (Sigma, USA) and following the standard steps for RNA extraction. RNA was reverse transcribed into cDNA according to the instructions of the kit (HiScript II 1st Strand cDNA Synthesis Kit, Vazyme, China), and the expression level of the target gene was detected by SYBR® Green Real-Time PCR (Vazyme, China). Primer sequences (Tsingke Biotechnology Co., Ltd., China) are presented in Table 1.

### 2.5. Western Blot

The cells were lysed in the radioimmunoprecipitation assay (RIPA) lysate of a total protein extraction kit (Bioswamp, China), and the protease inhibitor phenylmethylsulfonyl fluoride (PMSF) (Servicebio, China) and a cocktail (Servicebio, China) were added to obtain protein extracts. Then, 40 *μ*g of protein from each group was added to sodium dodecyl sulfate-polyacrylamide gel electrophoresis (SDS-PAGE) gels and transferred to polyvinylidene fluoride (PVDF) membranes. The membranes were incubated with primary antibodies against GSDME (#AB215191, Abcam), caspase-9 (#9502, Cell Signaling Technology), caspase-3 (#9662, Cell Signaling Technology), caspase-7 (#12827, Cell Signaling Technology), B-cell lymphoma-2 (Bcl-2) (#4223, Cell Signaling Technology), Bcl2-associated X (Bax) (#2774, Cell Signaling Technology), and GAPDH (#60004, Proteintech, China) overnight at 4°C. Then, the membranes were washed with Tris-buffered saline and tween 20 (TBST) and incubated with horseradish peroxidase- (HRP-) labelled secondary antibodies (#7074, Cell Signaling Technology). The protein bands were visualized with Image Lab software (Bio-Rad, USA).

### 2.6. Immunofluorescence

The slides of ARPE-19 cells and smears of Y79 and WERI-RB-1 cells were fixed with fixed fluid (95% alcohol) for 15 min and washed three times. The cells were permeabilized with 1% Triton X-100 for 10 min. After blocking with 5% bovine serum albumin (BSA) for 2 h, primary antibody (GSDME #13075, Proteintech, China) was added and incubated overnight at 4°C. The primary antibody was washed away, a secondary antibody (donkey anti-rabbit IgG-Alexa Fluor 594, Absin, China) was added for 2 h, and then, the secondary antibody was washed away. The cell nuclei were stained with 4,6-diamino-2-phenyl indole (DAPI) (Servicebio, China), and then the cells were filled with antifluorescence quencher, sealed into tablets, and observed microscopically.

### 2.7. Flow Cytometry

Phosphatidylserine (PS) is generally located inside the lipid bilayer of normal cell membranes. In the early stages of apoptosis, PS migrates to the lateral side from the inner membrane lipid bilayer. Annexin-V is a phospholipid-binding protein with a high affinity for PS that can bind to PS transferred to the outside of the cell membrane during early apoptosis. Thus, the fluorescent probe Annexin-V labelled with fluorescein isothiocyanate (FITC) can be used as a fluorescent probe for the detection of early apoptosis. Propidium iodide (PI) is a nucleic acid dye that cannot pass through normal membranes due to their barrier action but can dye the nucleus red in necrotic cells with altered membrane permeability during middle to late apoptosis. Thus, Annexin-V-FITC and PI double staining can differentiate cells in different apoptotic periods. After cell transfection with culture intervention for different times, cells were digested with trypsin (without ethylene diamine tetraacetic acid (EDTA)) and harvested by centrifugation at 1000 rpm×5 min at room temperature. The cells were resuspended in precooled PBS, and the supernatant was discarded by centrifugation at 1000 rpm×5 min. Ten microlitres of Annexin V-FITC and 5 *μ*l of PI were added to 85 *μ*l of 1× binding buffer and incubated with cells for 15 min at room temperature in the dark. The cells were resuspended in 400 *μ*l of 1× binding buffer and then detected on a flow cytometer (Beckman Coulter, CytoFLEX, USA) within 1 h. The laser was set to 488 nm, and the detectors were 525/40 BP and 585/42 BP; 10,000 cells were collected. Fluorescence intensity values were analysed by CytExpert software to calculate the apoptosis rate in each group.

### 2.8. Cell Transfection

Cells were seeded in six-well plates at 1 × 10^5^ cells/ml, and empty lentivirus (LV-CN) and LV-GSDME (GeneChem, China) with different multiplicities of infection (MOIs) (20, 40, 60, 80, and 100) were added to the medium. One millilitre of medium was added to six-well plates after 12 h, and the transfection efficiency was observed at 72 h by fluorescence microscopy. We found that the transfection efficiency was greater than 90% at an MOI of 100 (Extended Figure 1A); thus, an MOI of 100 was used for lentiviral transfection. The overexpression sequences of LV-GSDME are shown in Extended Data 1. Stable transfection was performed by adding 2 *μ*g/ml puromycin to the posttransfection media, and the fluid was changed once at approximately 3 days. A maintenance dose of puromycin (1 *μ*g/ml) (Servicebio, China) was added again.

### 2.9. Xenograft Tumor Model

Adult Balb/C nude mice (Beijing Vital River Laboratory Animal Technology Co., Ltd, China) weighing 20-25 g were used for approximately 6-7 weeks. The procedure was approved by the Guidelines for The Use of Animal Care and Laboratory Animals issued by Wuhan University. All mice were fed unified standard chow, had free access to food and drinking water, and were housed under a standard interior environment (20-25°C, 50-70% humidity). After the cells were mixed with matrix glue (#354234, Becton, Dickinson and Company) at a ratio of 1 : 1, 1 × 10^7^ cells were injected subcutaneously above the right thigh. The Balb/C nude mice were divided into four groups: the first group and the second group were inoculated with Y79 cells, the third group was inoculated with Y79 cells with stable transformation of LV-CN, and the fourth group was inoculated with Y79 cells with stable transformation of LV-GSDME. Tumor formation occurred approximately 2 weeks later. When the tumor was approximately 100 mm^3^, the second group was injected with 0.5 mg/kg decitabine (MedChemExpress, USA) 1 time/day for 7 days in total. The other groups were intraperitoneally injected with the same volume of saline. Seven days later, 50 mg/kg carboplatin was intraperitoneally injected three times a week. Tumor tissues were isolated and removed 2 weeks later under general anaesthesia. Tumor volume was estimated using the following formula: [width]^2^ × [length] × 1/_2_. The tumor was weighed, stored, fixed in 4% paraformaldehyde, and embedded in paraffin sections for TUNEL detection.

### 2.10. Enzyme-Linked Immunosorbent Assay

The cell supernatants were collected, and the level of lactate dehydrogenase (LDH) was measured using the Human LDH ELISA Kit (#HM10217 Bioswamp Wuhan, China). For the human LDH assay of the samples, purified human LDH antibody was used to coat microtiter plate wells to generate a solid-phase antibody, and then, LDH was added to the wells. The LDH antibody was combined with HRP for labelling, resulting in an antibody-antigen-enzyme-antibody complex. After washing completely, tetramethyl benzidine (TMB) substrate solution was added, the TMB substrate developed a blue colour under catalysis by the HRP enzyme, the reaction was terminated by the addition of a sulfuric acid solution, and the colour change was measured spectrophotometrically at a wavelength of 450 nm. The LDH concentration in the samples was then determined by comparing the optical densities (ODs) of the samples to the standard curve.

### 2.11. Statistical Analysis

All experiments were performed at least three times. Statistical analyses were performed using Student's *t* test with GraphPad Prism and Pearson chi-square test. All quantified data are expressed as the mean ± standard deviation (SD). Unless otherwise stated, ^∗^*P* < 0.05 was considered significant. Significant differences are indicated by ^∗∗^*P* < 0.01 and ^∗∗∗^*P* < 0.001, and ns indicates nonsignificant.

## 3. Results

### 3.1. GSDME Expression in Tumor Tissues and Peritumoral Normal Retinal Tissues in RB

**Figure 1:**
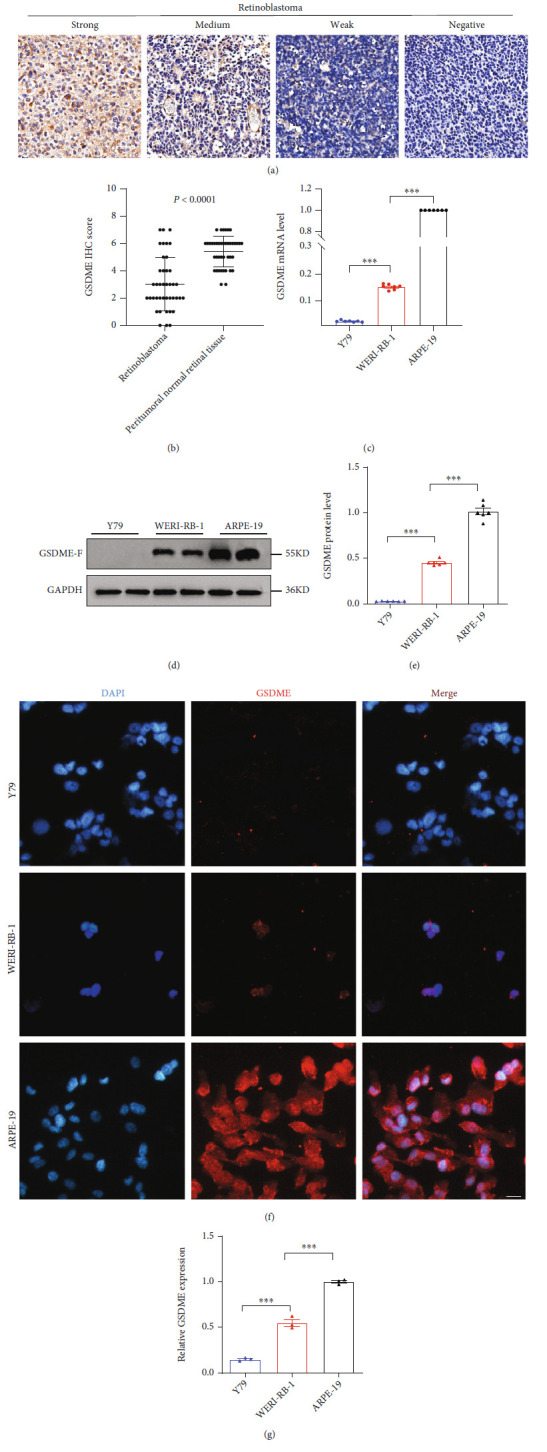
GSDME expression differences in human retinoblastoma. (a) Representative images of weak, medium, strong, and negative GSDME IHC staining. (b) Scatterplots of the average staining scores of GSDME expression in RB and peritumoral normal retinal tissues. (c) RT-PCR was applied to detect GSDME mRNA levels in the Y79, WERI-RB-1, and ARPE-19 cell lines. (d and e) The expression of GSDME protein was detected in the three cell lines using WB, and a quantitative map was drawn. (f and g) The expression of GSDME protein was detected in the three cell lines using immunofluorescence, and a quantitative map was drawn. Each experiment was repeated at least three times. Scale bar, 100 *μ*m.

First, we examined the expression of GSDME protein in RB patients by IHC assay and found that GSDME protein was significantly reduced in tumor tissue compared with peritumoral normal retinal tissue (Figures 1(a) and 1(b)). As shown in Table 2, the decrease in GSDME expression was significantly correlated with RB clinical tumor node metastasis (cTNM) (*P* = 0.0288), regional lymph node (N stage) (*P* = 0.0431), and pathological tumor node metastasis (pTNM) (*P* = 0.0425); however, no significant association was found between GSDME expression and sex. To further verify the relationship between GSDME and RB, quantitative real-time polymerase chain reaction (RT-PCR) results showed that the mRNA expression of GSDME in Y79 and WERI-RB-1 cells was significantly lower than that in ARPE-19 cells, and the mRNA expression of GSDME in Y79 cells was significantly lower than that in WERI-RB-1 cells (Figure 1(c)). Western blot (WB) analysis showed that Y79 cells had the lowest expression of GSDME protein, and the expression of GSDME protein in WERI-RB-1 cells was significantly lower than that in ARPE-19 cells (Figures 1(d) and 1(e)). The cellular immunofluorescence results were consistent with the WB results (Figures 1(f) and 1(g)). These data indicate that GSDME expression was reduced in human RB tissues and RB cell lines.

### 3.2. Carboplatin-Induced Cell Death with High GSDME Expression Switched Apoptosis to Pyroptosis

**Figure 2:**
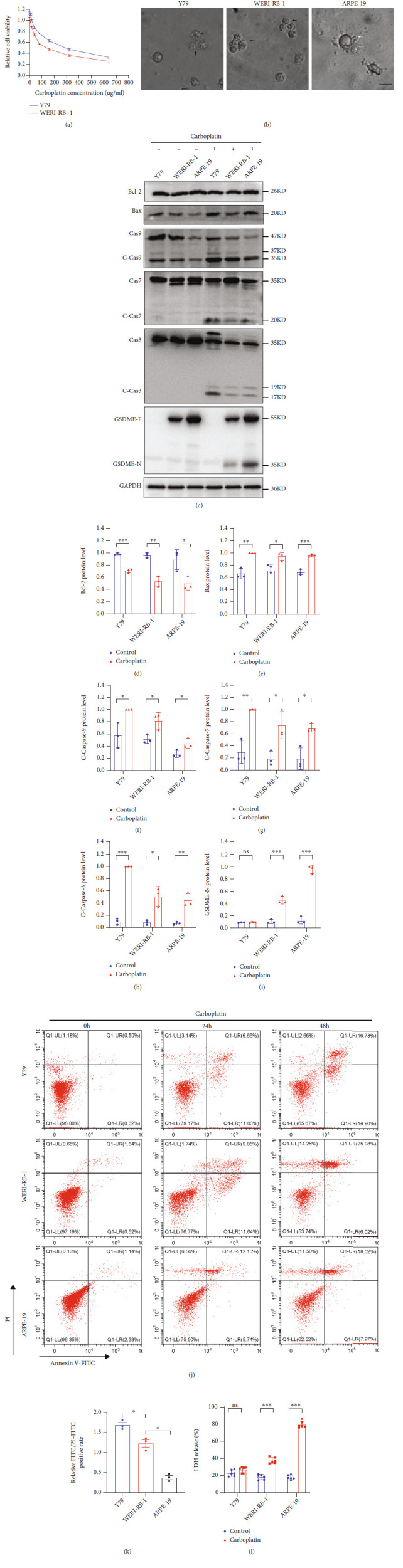
Carboplatin induces pyroptosis in cells with high GSDME expression. (a) CCK-8 assay of cell viability. (b) The death morphologies of the three cell lines were observed using an Olympus microscope; black arrows, apoptotic cells; white arrows, pyroptotic cells. (c–i) GSDME-N and pathway proteins were tested by WB in three different cell lines. (j and k) The FITC and PI staining rates of the three cell lines were determined by flow cytometry, and quantitative maps were drawn after treatment with carboplatin for 24 h. (l) LDH levels in the cell supernatant were detected by ELISA. Each experiment was repeated at least three times. Scale bar, 100 *μ*m.

GSDME has recently been considered a tumor suppressor that promotes tumor cell death by inducing pyroptosis. Carboplatin was used to induce the death of Y79 and WERI-RB-1 cells, and we found that the IC_50_ in Y79 cells was significantly higher than that in WERI-RB-1 cells (Figure 2(a) and Table 3). First, observation of the death morphology of the three cell lines showed that the main form of cell death was apoptosis in Y79 cells but pyroptosis in ARPE-19 cells, and some apoptosis occurred in WERI-RB-1 cells (Figure 2(b)). In the case of carboplatin-induced cell death in Y79, WERI-RB-1, and ARPE-19 cells, a decrease in Bcl-2, an increase in Bax, and significant increases in activated caspase-9, activated caspase-7, and caspase-3 were observed. GSDME-N was present in cells expressing GSDME protein (Figures 2(c)–2(i)). We performed flow cytometry to detect cell death. After treatment with carboplatin for 24 h, the ratio of FITC monostaining to FITC and PI double-positive staining was higher in Y79 cells than in WERI-RB-1 cells, and the ratio of FITC monostaining to FITC and PI double-positive staining was higher in WERI-RB-1 cells than in ARPE-19 cells (Figures 2(j) and 2(k)). A large amount of LDH was detected in the cell supernatant of WERI-RB-1 and ARPE-19 cells (Figure 2(l)). However, in this study, we found that cleavage-activated caspase-9 was also present in the WB of the normal group, but no activated caspase-7 or caspase-3 was observed downstream—a finding that requires further study.

### 3.3. Upregulation of GSDME Expression in Y79 and WERI-RB-1 Cells by Decitabine Resulted in Enhanced Sensitivity to Carboplatin

**Figure 3:**
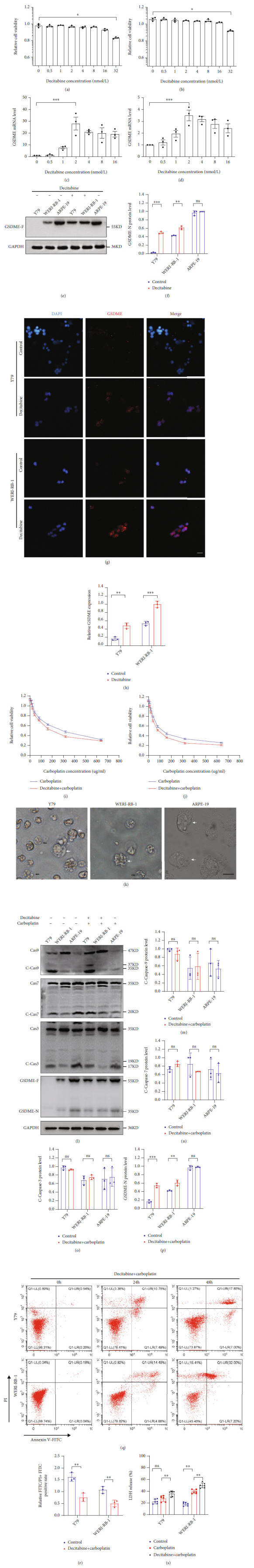
After GSDME expression was upregulated by decitabine, pyroptosis was increased. (a and b) The toxicity of decitabine to Y79 and WERI-RB-1 cells was detected by CCK-8 assay at different concentrations of decitabine. (c and d) The mRNA expression of GSDME was detected using RT-PCR after treatment with different concentrations of decitabine for 3 days (the cells were centrifuged, and decitabine was replaced every day). (e and f) The protein level of GSDME in cells was quantitatively analysed by western blot. (g and h) The protein level of GSDME in cells was detected using immunofluorescence. (i and j) CCK-8 assay of Y79 and WERI-RB-1 cell viability after treatment with carboplatin or decitabine plus carboplatin. (k) The death morphologies of the three cell lines were observed using an Olympus microscope. Y79 and WERI-RB-1 cells were treated with decitabine plus carboplatin; ARPE-19 cells were treated with decitabine; black arrows, apoptotic cells; white arrows, pyroptotic cells. (l–p) GSDME-N and its pathway proteins were detected by WB. (q and r) Flow cytometry showed the ratio of FITC monostaining to FITC and PI double-positive staining after treatment with decitabine plus carboplatin, and quantitative maps were drawn for carboplatin-treated cells after 24 h. (s) LDH in the cell supernatant was detected by ELISA. Each experiment was repeated at least three times. Scale bar, 100 *μ*m.

For Y79 and WERI-RB-1 cells with reduced GSDME expression, decitabine can upregulate GSDME expression to shift apoptosis to pyroptosis and increase the sensitivity of cells to carboplatin. When Y79 and WERI-RB-1 cells were treated with different concentrations of decitabine and when the drug concentration reached 32 nmol/l, cell viability was significantly reduced according to the CCK-8 assay (Figures 3(a) and 3(b)). RT-PCR revealed that the mRNA expression of GSDME was highest at the low concentration of 2 nmol/l decitabine (Figures 3(c) and 3(d)). The protein level of GSDME in cells treated with decitabine for 3 days was significantly higher than that in the untreated control group (Figures 3(e) and 3(f)), and the same results were observed with immunofluorescence (Figures 3(g) and 3(h)). The IC_50_ of Y79 and WERI-RB-1 cells was significantly reduced after treatment with decitabine plus carboplatin compared with the group not treated with decitabine (Figures 3(i) and 3(j) and Table 4). The proportion of cell death by pyroptosis was significantly increased in Y79 and WERI-RB-1 cells (Figure 3(k)). After decitabine addition, carboplatin increased GSDME-N, but no significant differences in the activated forms of caspase-9, caspase-7, and caspase-3 were found (Figures 3(l)–3(p)). Likewise, after treatment with decitabine for 3 days, flow cytometry showed that the ratio of FITC monostaining to FITC and PI double-positive staining declined in the carboplatin-treated group after 24 h compared with the group not treated with decitabine (Figures 3(q) and 3(r)). ELISA also detected an increase in LDH in the cell supernatant of the decitabine plus carboplatin groups (Figure 3(s)).

### 3.4. LV-GSDME Transfection of Y79 and WERI-RB-1 Cells Increased Pyroptosis

**Figure 4:**
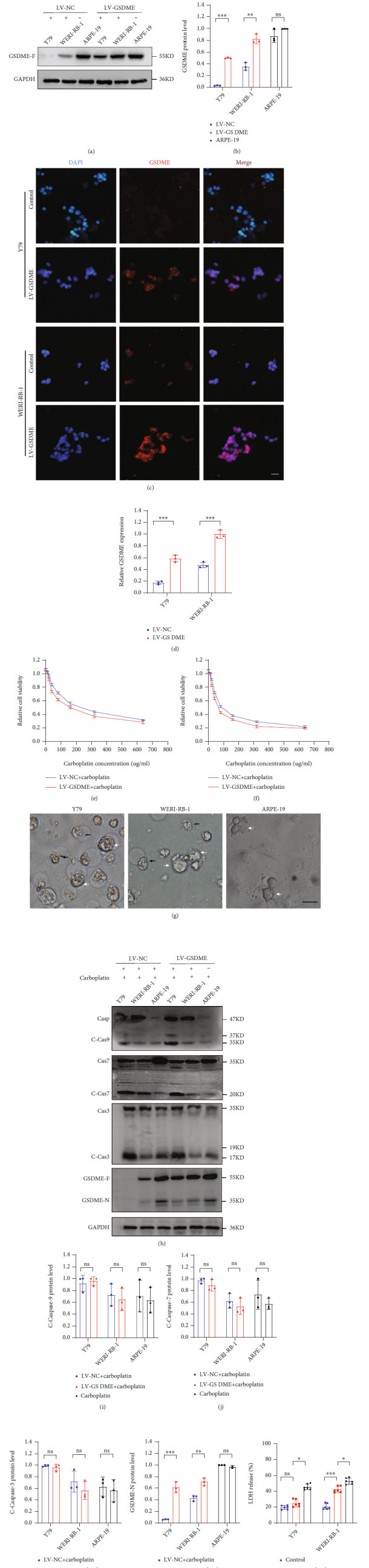
Pyroptosis increased in cell death forms after GSDME overexpression. (a and b) The expression level of GSDME protein was determined by WB. (c and d) The protein level of GSDME in cells was detected using immunofluorescence. (e and f) The IC_50_ of Y79 and WERI-RB-1 cells was detected using CCK-8. (g) The death morphologies of the three cell lines were observed using an Olympus microscope. Y79 and WERI-RB-1 cells were transfected with LV-GSDME; black arrows, apoptotic cells; white arrows, pyroptotic cells. (h–l) GSDME-N and its pathway proteins were detected by WB. (m) LDH in the cell supernatant was detected by ELISA. Each experiment was repeated at least three times. Scale bar, 100 *μ*m.

GSDME expression in Y79 and WERI-RB-1 cells was significantly reduced compared with that in normal retinal tissues. After transfection with LV-CN and LV-GSDME, GSDME protein expression was significantly increased in the LV-GSDME group (Figures 4(a) and 4(b)). Immunofluorescence also showed that the expression of GSDME protein was significantly increased after transfection with LV-GSDME (Figures 4(c) and 4(d)). Cell viability detection with CCK-8 showed that the IC_50_ in the LV-GSDME groups was significantly lower than that in the LV-CN groups (Figures 4(e) and 4(f) and Table 5). When GSDME was overexpressed by transfection with LV-GSDME, the mode of death in the Y79 and WERI-RB-1 cells was mainly pyroptosis (Figure 4(g)). Compared with the LV-NC group, the LV-GSDME group did not have significantly different levels of activated caspase-9, caspase-7, and caspase-3, but the GSDME-N level was significantly increased in the LV-GSDME group (Figures 4(h)–4(l)). ELISA detected a significant increase in LDH in the supernatant of the LV-GSDME groups (Figure 4(m)). Therefore, after the transfection with LV-GSDME, the results directly confirmed that the increase in GSDME protein expression promoted the transformation of the cell death mode from apoptosis to pyroptosis and reduced the IC_50_ of chemotherapeutic drugs.

### 3.5. Inhibition of Caspase-3 Activation and Switching Pyroptosis to Apoptosis

**Figure 5:**
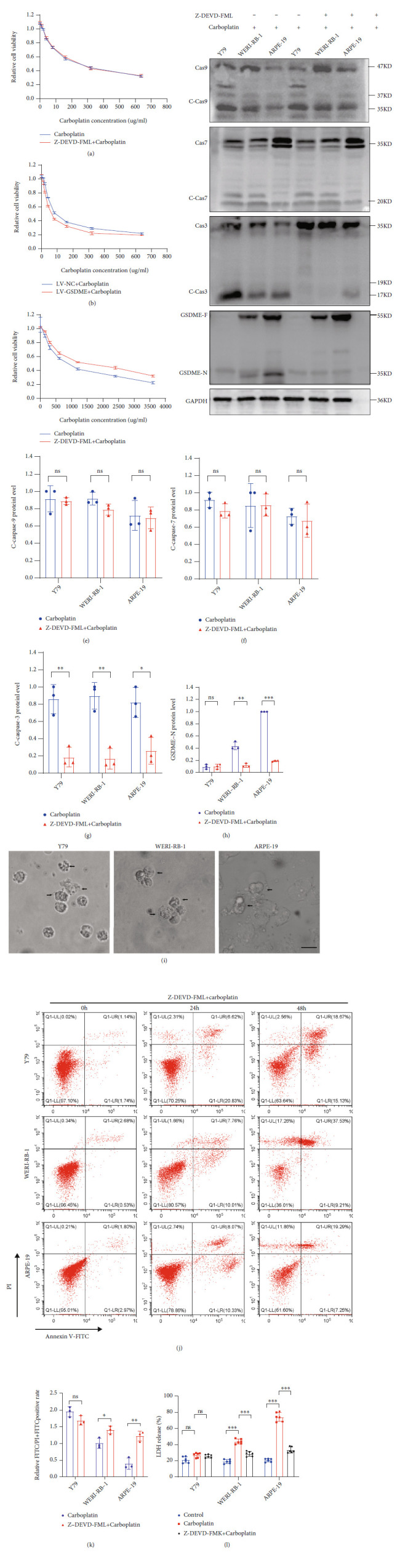
Inhibition of caspase-3 activation and pyroptosis was reduced in GSDME-expressing cells. (a–c) The IC_50_ values of three cell lines were detected using CCK-8. (d–h) The expression levels of GSDME-N and pathway proteins were determined by WB. (i) The death morphologies of the three cell lines were observed using an Olympus microscope after the three cell lines were treated to Z-DEVD-FML and carboplatin; black arrows, apoptotic cells. (j and k) Flow cytometry showed the ratio of FITC monostaining to FITC and PI double-positive staining after treatment with Z-DEVD-FML and carboplatin, and quantitative maps were drawn for carboplatin-treated cells after 24 h. (l) LDH in the cell supernatant was detected by ELISA. Each experiment was repeated at least three times. Scale bar, 100 *μ*m.

GSDME can be cleaved by activated caspase-3 to form functional GSDME-N. After the caspase-3 inhibitor Z-DEVD-FML (20 *μ*m/l) was added to Y79, WERI-RB-1, and ARPE-19 cells, carboplatin induced cell death simultaneously. No significant change in the IC_50_ in Y79 cells was found in the Z-DEVD-FML group compared to the non-Z-DEVD-FML group, but the IC_50_ of carboplatin in WERI-RB-1 and ARPE-19 cells was significantly increased (Figures 5(a)–5(c) and Table 6). WB detection of activated caspase-3 was significantly reduced, but caspase-9 and caspase-7 could still be cleaved to form active cleavage bodies, and no significant difference was found between the Z-DEVD-FML group addition group and the non-Z-DEVD-FML group, but in the Z-DEVD-FML group, GSDME-N (Figures 5(d)–5(h)) could not be detected, and the main form of cell death was apoptosis (Figure 5(i)). After carboplatin treatment for 24 h, the ratio of FITC monostaining to FITC and PI double-positive staining significantly increased in WERI-RB-1 and ARPE-19 cells, with no significant change in Y79 cells (Figures 5(j) and 5(k)). The LDH level was significantly lower in the Z-DEVD-FML group than in the non-Z-DEVD-FML group in WERI-RB-1 and ARPE-19 cells, with no significant change in Y79 cells (Figure 5(l)).

### 3.6. In Vivo Experiments Confirmed That Carboplatin Promoted the Death of Y79 Cells Treated with Decitabine and Transfected with LV-GSDME

**Figure 6:**
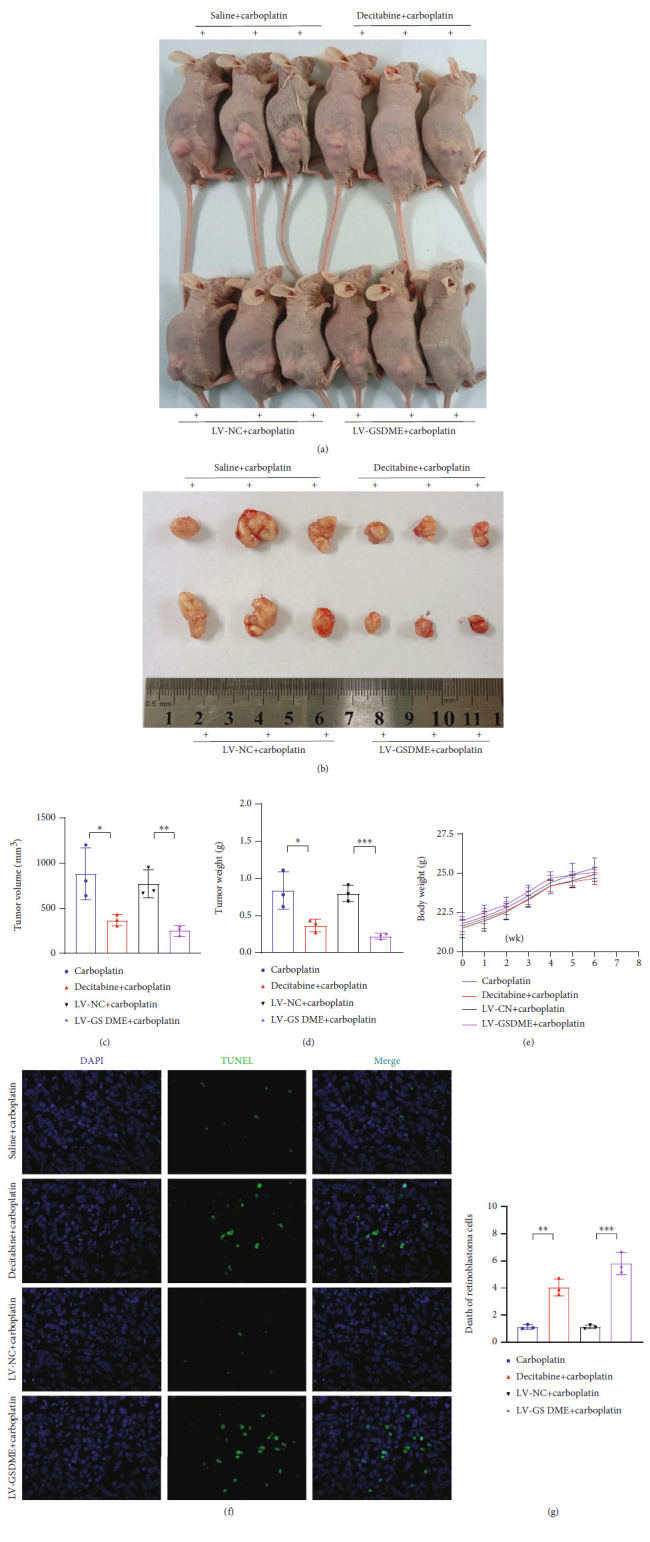
*In vivo* experiments confirmed that increased GSDME expression promoted cell death. (a–d) Images of three representative mice from the four groups are shown, and tumor sizes and tumor weights were measured. (e) After tumor cells were inoculated, the mouse body weight changes were detected. (f and g) TUNEL was used to detect cell mortality in the four groups. Each experiment was repeated at least three times. Scale bar, 100 *μ*m.

To investigate the efficacy of promoting GSDME expression *in vivo*, a xenograft tumor model was established. The tumor sizes and weights in the decitabine intervention and LV-GSDME-transfected groups were significantly lower than those in the control group (Figures 6(a)–6(d)). The body weights of the mice in the four groups did not change significantly, indicating that the mice had no side effects, such as marasmus, when the tumor cells were seeded (Figure 6(e)). Compared with the control group, the decitabine intervention and LV-GSDME-transfected groups had significantly more cell death based on the TUNEL assay (Figures 6(f) and 6(g)). Therefore, these data confirmed that at the same drug dose, the decitabine intervention and LV-GSDME transfection had stronger suppressive effects on the tumors *in vivo*.

## 4. Discussion

Retinoblastoma is mainly caused by mutations in the retinoblastoma protein gene (RB gene), a tumor suppressor gene. Mutations are categorized as germ cell mutations and somatic mutations. Approximately 40% of cases can be passed on to the next generation, with a small percentage involving activation of the protooncogene [28–32]. In cases of inactivation of the RB gene, the introduction of the normal RB gene into retinoblasts still cannot prevent tumor progression [33]. Therefore, for RB patients who cannot be effectively treated, enucleation is the most important treatment method. However, in developing countries, due to limited application of early screening and the inability of infants and young children to describe their symptoms, the disease is generally not discovered by parents until strabismus and leukocoria occur [34, 35]. At this point, most of the tumors are in the advanced stage, and metastasis, the leading cause of death in RB patients, has already occurred [34, 35]. In these cases, only drug treatment can be provided. With the improvement in chemotherapeutic drugs, RB treatment has markedly improved. However, the main problem with drug treatment is the likelihood of drug resistance. Therefore, studies of drug resistance are very important.

GSDME is considered a tumor suppressor, and its presence is negatively correlated with tumor viability. Its main function is to induce pyroptosis in cells [36]. Pyroptosis has been reported to be very important to the antitumor activity of chemotherapeutic drugs. The expression of GSDME mRNA in the etoposide-resistant human melanoma cell line MeWo-Eto-1 was found to be significantly lower than that in its parent nonresistant human melanoma cell line MeWo [37]. However, the sensitivity to etoposide increased by approximately 30-35% after GSDME expression was increased in MeWo-Eto-1 cells [37]. Miltirone extracted from the root of *Salvia miltiorrhiza* Bunge has antitumor activity through the induction of GSDME activation [38]. Decreased GSDME expression increases the acquired drug resistance of melanoma cells [37]. Based on these reports, we hypothesized that the level of GSDME expression was related to the prognosis of RB. Therefore, in our study, we found that GSDME was differentially expressed in human RB tissues and that GSDME expression was inversely proportional to tumor stage. Further supporting this finding, we confirmed that GSDME expression was significantly reduced in the RB cell lines Y79 and WERI-RB-1 compared with the normal retinal pigment epithelial cell line ARPE-19. Moreover, Y79 cells with lower GSDME expression had lower sensitivity to carboplatin, and the IC_50_ in Y79 cells was significantly higher than that in WERI-RB-1 cells, indicating that the increase in GSDME expression promotes cell death and reduces the cell survival rate.

As a tumor suppressor gene, GSDME inactivation is mainly caused by methylation of its promoter. Due to promoter hypermethylation, GSDME expression is downregulated in breast cancer, gastric cancer, oesophageal cancer, and colorectal cancer [22, 23, 39–43]. In the mouse colon cancer cell line CT-26 and the mouse breast cancer cell line 4 T-1 with reduced GSDME expression, GSDME expression was significantly increased after decitabine intervention [24, 44]. Similarly, after low-dose decitabine treatment of Y79 and WERI-RB-1 cells, GSDME expression was significantly increased, but low-dose decitabine did not increase cell mortality. The IC_50_ of carboplatin that induced cell death was significantly lower in the decitabine groups. Increased GSDME expression increased the sensitivity of cells to carboplatin, and this increase correlated with the form of cell death. The increase in GSDME was accompanied by an increase in pyroptosis at the IC_50_ of carboplatin-induced cell death, confirming that GSDME expression was positively correlated with drug sensitivity for carboplatin-induced cell death.

To further investigate whether the increase in GSDME increases drug sensitivity, we transfected cells with LV-GSDME. We found that GSDME expression was significantly increased, and the carboplatin IC_50_ for Y79 and WERI-RB-1 cells was significantly decreased. Cell pyroptosis and LDH release were significantly increased.

**Figure 7:**
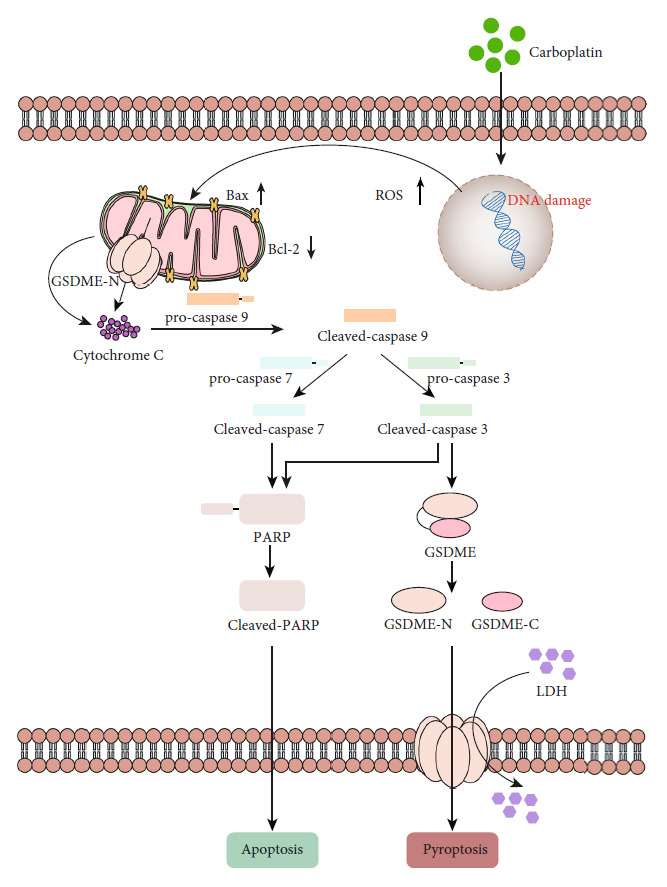
Schematic diagram of carboplatin-induced cell death in GSDME-expressing cells.

Drug sensitivity was enhanced by increased pyroptosis. Increasing GSDME expression can reduce the required dosage of chemotherapy drugs. First, when carboplatin enters the cell, DNA damage increases internal stress in cells, inhibits Bcl-2, increases of Bax, and induces changes in mitochondrial membrane potential. These effects lead to cytochrome C release, which activates caspase-9. Activated caspase-9 further activates downstream caspase-7 and caspase-3, and activated caspase-7 and caspase-3 cleave PARP to inactivate it and induce cell apoptosis [21]. However, in the presence of GSDME, activated caspase-3 preferentially cleaves GSDME and forms GSDME-N, which is inserted into the cell membrane to induce pyroptosis. Second, GSDME-N was also confirmed to insert into the mitochondrial membrane of cells to augment the release of cytochrome C and induce cell death (Figure 7). GSDMA, which is also a member of the GSDM family, has been shown to induce a mitochondrial permeability shift through insertion into the mitochondrial membrane [45, 46]. Studies have also shown that GSDMD-N can destroy the mitochondrial membrane potential, leading to mitochondrial changes before rupture of the cell membrane [47]. In addition, GSDME-N was inserted into the mitochondrial membrane of *Saccharomyces cerevisiae*, which also led to elevated reactive oxygen species levels, oxidative stress, and cell death [48, 49]. Z-DEVD-FML inhibited caspase-3 activation, GSDME-N formation, and the occurrence of pyroptosis. After treatment with Z-DEVD-FML, the IC_50_ of carboplatin in Y79 cells was not significantly different from that in untreated cells; however, the IC_50_ of carboplatin in WERI-RB-1 and ARPE-19 cells was significantly increased. Subsequently, pyroptosis and LDH release were also significantly reduced. *In vitro* studies have confirmed that increased GSDME expression can promote the occurrence of pyroptosis and increase sensitivity to chemotherapeutic drugs. This finding was confirmed by the xenograft tumor model *in vivo*.

Our study found that GSDME expression was positively correlated with the prognosis of RB. GSDME increased drug sensitivity by inducing pyroptosis. The reason for this effect was that many chemotherapeutic drugs cannot induce apoptosis in tumor cells. Carboplatin promotes the degradation of DNA repair enzymes after DNA damage, leading to cell apoptosis. However, after carboplatin intervention, tumors form a new DNA repair mechanism, and the proapoptotic capacity of carboplatin is reduced, resulting in cell resistance to carboplatin [6]. However, pyroptosis does not depend on this mechanism. GSDME activation leads to the formation of pores in the cell membrane, which causes extracellular fluid to enter the cell; as a result, the cells swell and die. In the presence of GSDME, caspase-3 preferentially activates GSDME, leading to pyroptosis. Moreover, GSDME-N forms pores in not only the cell membrane but also the mitochondrial membrane, leading to changes in mitochondrial membrane potential and depolarization and causing further release of cytochrome C and cell death caused by GSDME-N [21], which allows a reduction in the use of chemotherapeutic drugs (Figure 7).

## 5. Conclusion

This study demonstrates that GSDME plays important roles in the regulation of the carboplatin sensitivity in RB cells. These observations indicate that GSDME represents a potential target for overcoming chemoresistance in RB by inducing pyroptosis, creating a positive feedback mechanism to further promote the death of tumor cells. Pyroptosis will be an alternative mode death of many drug-resistant tumor cells. At the same time, high GSDME expression was found in RB vascular endothelial cells in paraffin sections of human RB tissues, and for tumor treatment, whether tumor cell death can be further promoted by inducing pyroptosis of vascular endothelial cells will be our next research direction.

## Figures and Tables

**Table 1:** Primer sequences.

Gene (human)	Sequence
*β*-Actin	
Forward	5′-GAAGTCCCTCACCCTCCCAA-3′
Reverse	5′-GGCATGGACGCGACCA-3′
GSDME	
Forward	5′-GATCTCTGAGCACATGCAGGTC-3′
Reverse	5′-GTTGGAGTCCTTGGTGACATTCC-3′

**Table 2:** Association of GSDME expression with clinicopathological characteristics of RB.

Clinicopathological features				
	GSDME expression			
	Number	Low	High	*P* value
Sex				0.5033
Male	18	11	7	
Female	23	17	6	
Clinical tumor node metastasis (cTNM)				0.0288
T1-T2	12	5	7	
T3-T4	29	23	6	
N stage				0.0431
N0	21	11	10	
N1-N3	20	17	3	
Pathological tumor node metastasis (pTNM)				0.0425
T1-T2	18	9	9	
T3-T4	23	19	4	

**Table 3:** IC_50_ values and statistical analyses of carboplatin treatments in RB cell lines.

	IC_50_
	Carboplatin
Y79	121.9 (99.04 to 151.3)
WERI-RB-1	65.53 (45.89 to 77.18)

IC_50_ values show the carboplatin concentration (*μ*g/ml) (concentration, mean (95% confidence intervals)).

**Table 4:** IC_50_ values and statistical analyses of decitabine and carboplatin treatments in RB cell lines.

IC_50_		
	Carboplatin	Decitabine+carboplatin
Y79	125.9 (105.2 to 151.5)	80.93 (66.07 to 99.82)
WERI-RB-1	72.98 (61.59 to 89.91)	54.34 (38.78 to 60.64)

IC_50_ values show the decitabine concentration (2 nmol/l) and carboplatin concentration (*μ*g/ml) (concentration, mean (95% confidence intervals)).

**Table 5:** IC_50_ values and statistical analyses of LV-CN plus carboplatin and LV-GSDME plus carboplatin treatments in RB cell lines.

IC_50_		
	LV-CN+carboplatin	LV-GSDME+carboplatin
Y79	120.0(103.1 to 140.3)	75.83(62.22 to 93.10)
WERI-RB-1	70.46(56.43 to 88.76)	46.60(29.58 to 55.11)

IC_50_ values show carboplatin concentration (*μ*g/ml) (concentration, mean (95% confidence intervals)).

**Table 6:** IC_50_ values and statistical analyses of carboplatin and Z-DEVD-FML plus carboplatin treatments in RB cell lines.

IC_50_		
	Carboplatin	Z-DEVD-FML+carboplatin
Y79	123.6 (107.5 to 142.7)	129.2 (113.5 to 147.5)
WERI-RB-1	69.68 (56.93 to 85.90)	125.5 (108.7 to 145.4)
ARPE-19	579.8 (438.1 to 783.8)	1030 (795.3 to 1366)

IC_50_ values show the carboplatin concentration (*μ*g/ml) (concentration, mean (95% confidence intervals)).

## Data Availability

The data used to support the findings of this study are available from the corresponding author upon request.

## Supplementary Materials

Supplementary 1 Extended Figure 1: (A) at an MOI of 100, the transfection efficiency of Y79 and WERI-RB-1 cells is shown by green fluorescence.

Supplementary 2 Extended Data 1: overexpressed sequences of GSDME.
