# Supplementary material for: GSDME Increases Chemotherapeutic Drug Sensitivity by Inducing Pyroptosis in Retinoblastoma Cells
Source: Oxid Med Cell Longev. 2022 Mar 29;2022:2371807. doi: 10.1155/2022/2371807 (PMC9035765; doi:10.1155/2022/2371807)
Supplement: Supplementary 1 — Extended Figure 1: (A) at an MOI of 100, the transfection efficiency of Y79 and WERI-RB-1 cells is shown by green fluorescence. [file 2371807.f1.docx]

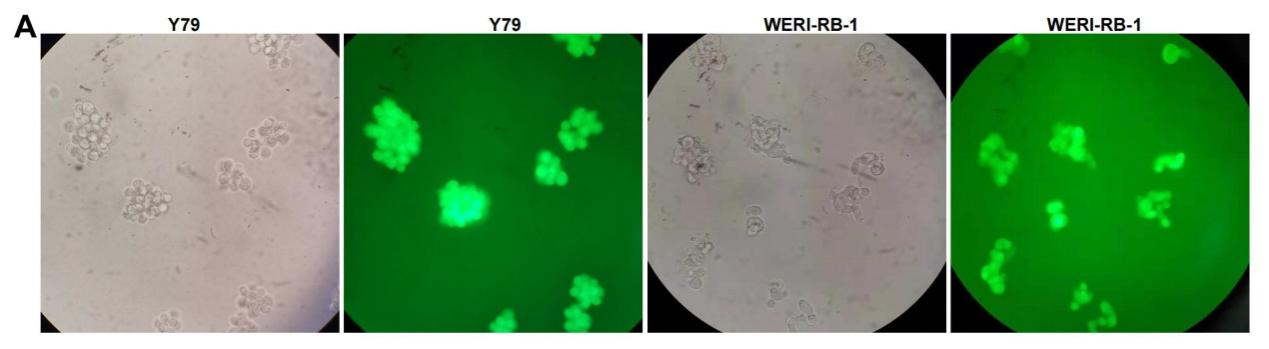


**Extended figure 1.(A)** At MOI 100, the transfection efficiency of Y79 and WERI-RB-1 cells was shown by green fluorescence.
