# Supplementary material for: GSDME Increases Chemotherapeutic Drug Sensitivity by Inducing Pyroptosis in Retinoblastoma Cells
Source: Oxid Med Cell Longev. 2022 Mar 29;2022:2371807. doi: 10.1155/2022/2371807 (PMC9035765; doi:10.1155/2022/2371807)
Supplement: Supplementary 2 — Extended Data 1: overexpressed sequences of GSDME. [file 2371807.f2.docx]

**Extended data 1.**Overexpressed sequences of GSDME：CGCTAATTCTGGCCGTTTTTGGCTTTTTTGTTAGACGAAGCTTGGGCTGCAGGTCGACTCTAGAGGATCCCGCCACCATGTTTGCCAAAGCAACCAGGAATTTTCTTAGAGAAGTTGATGCTGATGGTGACCTGATTGCAGTATCAAATCTGAATGACTCTGATAAGTTACAGCTTCTAAGTCTGGTGACAAAAAAGAAGAGATTCTGGTGCTGGCAGAGACCCAAGTACCAGTTTTTATCCCTCACCCTTGGCGATGTACTCATAGAAGACCAATTTCCGAGTCCAGTGGTCGTGGAGTCGGACTTTGTGAAATACGAGGGCAAGTTTGCAAACCACGTGAGTGGAACCCTGGAGACTGCACTGGGGAAGGTCAAGCTGAACCTGGGGGGCAGCAGCCGCGTAGAGAGCCAGTCTTCATTTGGAACCCTGAGGAAGCAGGAGGTGGATTTGCAGCAGCTCATCAGAGACTCTGCCGAGAGAACAATAAATCTGAGAAACCCTGTGCTCCAGCAGGTGCTGGAAGGAAGGAATGAGGTCCTGTGCGTTTTGACACAGAAGATCACGACGATGCAGAAGTGTGTGATCTCTGAGCACATGCAGGTCGAGGAGAAGTGTGGTGGCATCGTGGGCATCCAGACCAAGACGGTGCAGGTGTCAGCGACGGAGGATGGGAATGTCACCAAGGACTCCAACGTGGTGCTGGAGATCCCAGCTGCCACCACCATTGCCTACGGTGTCATTGAGTTATACGTGAAACTGGACGGCCAGTTCGAGTTCTGCCTTCTCCGAGGGAAGCAAGGTGGCTTCGAGAACAAGAAGAGAATTGACTCTGTCTACCTGGACCCCCTGGTCTTTCGAGAGTTTGCATTCATAGACATGCCAGATGCTGCGCATGGGATATCTTCCCAGGATGGACCATTAAGTGTTTTAAAGCAAGCGACCCTGCTCCTGGAGAGGAATTTCCATCCATTTGCGGAGCTGCCTGAGCCACAACAGACAGCTTTGAGTGACATCTTCCAGGCGGTCCTATTTGATGATGAACTACTCATGGTCCTGGAACCAGTGTGCGATGACCTGGTCAGCGGCCTCTCGCCCACAGTGGCGGTGCTGGGGGAGCTGAAGCCCCGGCAGCAGCAGGACCTTGTGGCCTTCCTGCAGCTGGTGGGGTGCAGCTTACAGGGTGGGTGTCCGGGCCCCGAGGATGCAGGCAGCAAGCAGCTGTTTATGACAGCCTACTTCTTGGTCAGTGCCCTCGCAGAAATGCCAGATAGCGCAGCAGCTCTGCTGGGCACTTGCTGCAAACTCCAGATCATTCCCACACTGTGCCACTTGCTTCGTGCTCTGTCTGATGATGGAGTATCTGATCTTGAAGACCCAACCTTGACTCCCCTGAAAGATACAGAAAGGTTTGGGATTGTGCAGCGCTTGTTTGCCTCAGCTGACATTAGTCTGGAGAGACTGAAGTCATCTGTGAAAGCTGTCATTCTGAAGGACTCTAAAGTCTTCCCACTGCTTCTTTGTATAACCCTGAATGGACTCTGTGCTTTAGGCAGAGAACATTCAGGTATGGACTACAAGGATGACGA.
